# Microbial keystone taxa and metabolic signatures in centenarians regulate intestinal homeostasis during aging

**DOI:** 10.1002/imt2.70134

**Published:** 2026-05-19

**Authors:** Wei‐Chuan Lin, Cui Zhang, He‐Hua Lei, Zheng Cao, Xin Gao, Wen‐Kai Yu, Xin‐Zhi Li, Qing‐Wei Xiang, Zhi‐Wen Zhang, Shi‐Fu Pang, Wei‐Fei Luo, Deng‐Hui Xie, Li‐Min Zhang, Gang Chen

**Affiliations:** ^1^ State Key Laboratory of Magnetic Resonance and Imaging, National Centre for Magnetic Resonance in Wuhan, Innovation Academy of Precision Measurement Science and Technology Chinese Academy of Sciences (CAS) Wuhan China; ^2^ University of Chinese Academy of Sciences Beijing China; ^3^ School of Pharmacy, Faculty of Medicine, Laboratory for Drug Discovery from Natural Resource, State Key Laboratory of Quality Research in Chinese Medicine Macau University of Science and Technology Macao China; ^4^ Hubei Shizhen Laboratory, Department of Geriatrics & Department of Orthopedic Surgery Hubei Provincial Hospital of Traditional Chinese Medicine (Affiliated Hospital of Hubei University of Chinese Medicine) Wuhan China; ^5^ Guangxi Key Laboratory of Longevity Science and Technology AIage Life Science Corporation Ltd. Nanning China; ^6^ Institute of Biological Science and Technology Guangxi Academy of Sciences Nanning China; ^7^ Department of Joint Surgery, Center for Orthopaedic Surgery The Third Affiliated Hospital of Southern Medical University Guangzhou China

**Keywords:** centenarians, Clostridium scindens, indole‐3‐acetic acid, keystone taxa, microbe–host interaction

## Abstract

Microbial networks and keystone taxa play pivotal roles in maintaining gut microecological stability and host homeostasis, irrespective of their abundance. However, most previous studies of aging‐associated gut microbiota have relied on abundance‐based analyses, largely overlooking microbial networks and microbe‐host interactions. Here, we employed a co‐occurrence network approach to identify keystone taxa during aging in humans and mice. We found that centenarians harbor distinctive keystone taxa dominated by members of *Clostridium*, of which *Clostridium scindens* (*C. scindens*) can significantly enhance microbial network stability, probably contributing to longevity and reduced susceptibility to age‐related diseases. Mechanistically, *C. scindens* produces indole‐3‐acetic acid (IAA) from tryptophan via the enzymes amidase (AMIE) and aldehyde dehydrogenase (ALDH). Oral administration of either *C. scindens* or IAA effectively mitigates intestinal aging by restoring gut barrier dysfunction in aged mice. Further analysis revealed that *C. scindens*‐derived IAA restores intestinal dysfunction through activation of aryl hydrocarbon receptor (AHR) signaling, leading to upregulation of intestinal CLDN10, a key tight junction protein. Structurally, IAA enhances *Claudin*‐*10* transcription by promoting AHR binding to its promoter region. Our findings provide new insights into the characterization of microbial networks in centenarians and highlight that *C. scindens* and IAA may contribute to healthy longevity by promoting gut microecological stability and host homeostasis.

## Highlights

- A co‐occurrence network analysis identifies keystone taxa dominated by members of *Clostridium* in centenarians.
- *Clostridium scindens* enhances microbial network stability, probably contributing to longevity and reduced susceptibility to age‐related diseases.
- *Clostridium scindens* produces indole‐3‐acetic acid (IAA) from tryptophan metabolism via its own enzymes amidase (AMIE) and aldehyde dehydrogenase (ALDH).
- *Clostridium scindens‐*derived IAA promotes intestinal homeostasis by facilitating aryl hydrocarbon receptor (AHR)‐mediated CLDN10 signaling in aged mice.

## Graphical abstract

*Clostridium scindens* and its metabolite indole‐3‐acetic acid (IAA) are identified as a keystone taxon and metabolic signature in centenarians. *Clostridium scindens* enhances microbial network stability and produces IAA via its own amidase (AMIE) and aldehyde dehydrogenase (ALDH), thus promoting intestinal homeostasis by activating the aryl hydrocarbon receptor (AHR)‐mediated *Claudin*‐*10* signaling in aged mice.

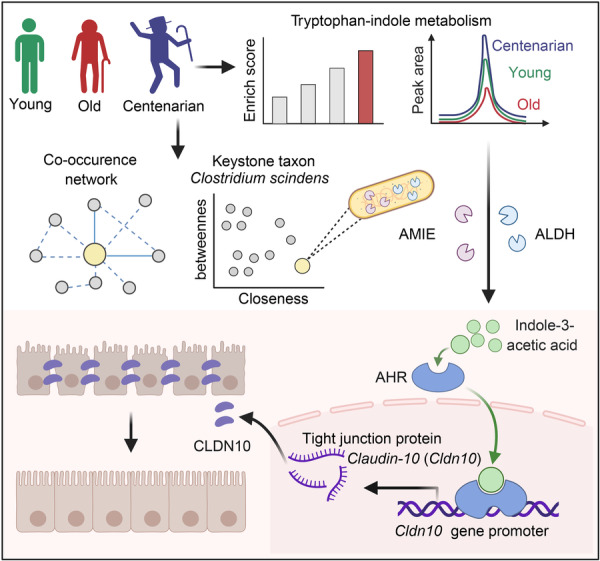

## INTRODUCTION

The World Health Organization (WHO) predicts that the global population aged 60 years old and over will reach 2.1 billion by 2050, thus posing unprecedented challenges to healthcare and society [1]. Geroscience, an emerging interdisciplinary science seeking to understand longevity and aging mechanisms, operates on the principle that aging is the biggest risk factor for many age‐related diseases, such as diabetes, cardiovascular disease, cancer, and neurodegenerative disorders [2, 3, 4]. Intestinal dysbiosis is one of the recently proposed 14 primary hallmarks of aging driving the aging process [4]. Distinct shifts occur in the diversity, composition, and functionality of human gut microbiota during aging, and maintaining a relative “youthful” microbial community has been shown to mitigate age‐related decline [5, 6, 7]. Moreover, fecal microbiota transplantation experiments demonstrated that colonization young microbiota to relatively older individuals can restore aging‐related phenotypes in animal models and even extend their lifespan [6, 7]. Meanwhile, the metabolic pathways involved in aging hallmarks, especially crosstalk between gut microbiota and host organs, closely contribute to aging and aging‐related disorders [8, 9]. It is therefore essential for identifying robust biomarkers of dysbiosis during aging, which will advance our understanding of human longevity and guide precision geromedicine for age‐related diseases interventions.

Increasing evidence has shown that the human gut microbiota, strongly attributed to host genetics, lifestyle, and environmental factors, plays a crucial role in human health, many chronic diseases, and aging [10, 11]. As people become older, the gut microbiome displays declined diversity accompanied by fewer beneficial microbes and more pathogenic microbes, suggesting that the gut microbiome and functional signatures are associated with longevity. Centenarians have a decreased susceptibility to aging‐associated chronic inflammation and infectious diseases [12, 13], which represents an excellent status of longevity at least partly linking to the functional capacity of their gut microbiome. Previous studies identified a reduction in the opportunistic or pathogenic microbiota and enrichment of beneficial microorganisms, including *Lactobacillus*, *Akkermansia*, and *Bifidobacterium*, in the centenarian group [6, 7]. Regarding the identification of potential microbial biomarkers of longevity and aging, centenarians, who are individuals aged 100 years or older, largely escaped major chronic diseases, providing a natural model of exceptional longevity [14, 15]. Although the microbiota composition of centenarians has been shown to be important in healthy aging, numerous cross‐sectional studies have relied on differential abundance analyses, primarily cataloguing taxa that are enriched or depleted to determine putative biomarkers [16, 17]. These approaches only concern microbial abundance without considering their functions and interactions across species. In fact, microbial communities contain keystone taxa, which drive community composition and function irrelevant to their abundance [18]. More importantly, keystone taxa exert disproportionate impacts on community stability and facilitate structural and functional outcomes beyond what their abundance would predict [19]. In microbial ecosystems, keystone taxa may be rare but highly associated with multiple species that shape resilience and homeostasis [20]. It is known that gut dysbiosis in microbial diversity and composition is one of the outstanding characteristics of aging [21, 22, 23]. However, the microbial networks and keystone taxa within the gut microbiota ecosystem for the identification of biomarkers during aging remain elusive. Hence, co‐occurrence patterns coupled with microbial networks can be used to statistically identify keystone taxa of human microbiome with age.

In addition to the gut microbiota, its metabolites, such as short‐chain fatty acids (SCFAs), bile acids, and indole metabolites, are closely linked to aging and longevity due to their distinctive physiological functions [24, 25, 26]. For instance, a previous study revealed that centenarians are enriched in bacteria producing isoallolithocholic acid (iso‐LCA), a secondary bile acid capable of inhibiting multidrug‐resistant pathogens such as *Clostridioides difficile* and *Enterococcus faecium*, which reduced infection risk to maintain gut homeostasis [27]. The integrity of the intestinal epithelial barrier progressively deteriorates with age and is considered an evolutionarily conserved hallmark of aging observed across worms, flies, rodents, and humans [28]. It is maintained by proteins of adhesive and tight junctions (AJs and TJs), including zonula occludens (ZO), claudins (CLDN), and occludin (OCLDN) [29, 30]. In recent years, the gut microbiota and microbiota‐derived metabolites have been shown to contribute to signaling pathways and processes of aging and aging‐related diseases. Previous studies showed that *Bacteroides fragilis*‐derived 3‐phenylpropionic acid upregulated both mRNA and protein levels of intestinal ZO‐1 via activating AHR signaling to preserve gut homeostasis [31]. Tryptophan metabolite 5‐methoxyindoleacetic acid has been found to enrich in the gut of centenarians and can be used to suppress inflammation‐induced cellular senescence [32]. In view of the close connection between gut homeostasis and aging, we hypothesized that centenarian‐associated microbial keystone taxa and metabolic signatures may highly contribute to longevity via the maintenance of gut barrier integrity.

In this study, we systematically profiled the gut microbial communities and metabolic landscapes across three human cohorts involving healthy individuals with different ages. By applying ecological network analysis, we identified interaction‐driven microbial keystone taxa rather than solely relying on differential abundance. Integrative multi‐omics approaches were subsequently performed to link these keystone species with centenarian‐enriched metabolic signatures. In vivo and in vitro functional validation was eventually carried out to investigate whether and how taxa‐metabolite interactions contribute to intestinal homeostasis during aging. Collectively, this longitudinal study highlights that microbial keystone taxa and metabolic signatures of centenarians are important for host homeostasis and longevity.

## RESULTS

### Identification of microbial characteristics and keystone taxa in centenarians

**Figure 1:**
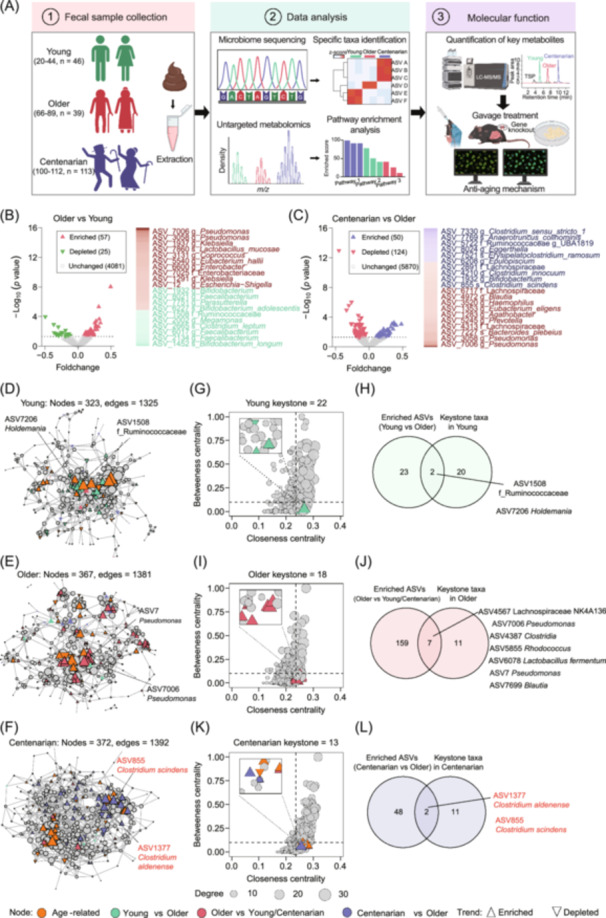
Identification of keystone taxa in the gut microbiome of centenarians. (A) Fecal samples collection and study workflow. (B, C) Differential amplicon sequence variants (ASVs) between centenarian or young and older group. The ASVs with FoldChange > 0 and −Log_10_ (*p*‐value) > 1.3 were selected as different ASVs. Red represents the ASVs enriched in the gut of older group, while green and purple represent the ASVs enriched in the gut of young and centenarian group, respectively. Unchanged ASVs were colored with gray. (D–F) Co‐occurrence network of gut bacterial community. Node size represents the degree of ASVs in network. Node color represents differential ASVs in young (green, Young vs. Older), older (red, Older vs. Young or Centenarian), and centenarian (purple, Centenarian vs. Older) groups. Orange node represents ASVs showed age‐related changes in their abundance. Node shape represents enriched (upper triangle), depleted (inverted triangle), unchanged (dot) ASVs. Only robust correlations in which |*r*| (Pearson's correlation coefficient) was >0.3 and *p* (Pearson significance coefficient) was <0.01 were visualized. Keystone taxa analysis (G) and their interactions with enriched ASVs in the young (H). Keystone taxa were ASVs with degree > 9, closeness centrality > 0.235, and betweenness centrality <0.10. (I, J) As in (G, H), for older group. (K, L) As in (G, H), for the centenarian group.

To investigate the taxonomic compositional and functional profiles of human microbiota, we recruited a cohort of three age groups: young (20–44 years old, *n* = 46), older (66–89 years old, *n* = 39), and centenarians (100–112 years old, *n* = 113) (Figure 1A). After quality control, a total of 198 qualified fecal samples were used to extract microbial DNA for DNA library construction and 16S rRNA gene amplicon sequencing. All sequences were annotated into 8265 amplicon sequence variants (ASVs). Although no significant differences were observed in α‐diversity among the three groups (Figure S1A), non‐metric multidimensional scaling (NMDS) based on ASV composition revealed significant age‐associated separations with distinct compositional dissimilarity (Figure S1B–E). In addition, permutational multivariate variance analysis showed that age instead of sex, BMI, and diet, etc., was the main factor relating to gut bacterial community (Table S1; *R*² = 0.02245, *F* = 2.3042, *p* = 0.001).

To further analyze these compositional shifts, “featured ASVs” as taxa were defined and uniquely enriched or depleted in a specific age group in comparison with others. These featured ASVs were classified into four categories: age‐related ASVs showing progressive trajectories across age groups, and group‐specific ASVs enriched in the young, older, or centenarian group, respectively. Based on this classification, we identified a total of 54 ASVs with progressive changes during aging (gradually increasing, *N* = 36; gradually decreasing, *N* = 18), 15 ASVs that were enriched (*N* = 8) or depleted (*N* = 7) in the young group, 14 ASVs that were enriched (*N* = 3) or depleted (*N* = 11) in the older group, and 41 ASVs uniquely associated with centenarians (enriched, *N* = 27; depleted, *N* = 14) (Figure S1F). Specifically, age‐related enrichment was dominated by ASVs belonging to *Clostridium sensu stricto 1*, *Turicibacter*, *Intestinibacter*, and *Klebsiella*, whereas taxa such as *Blautia*, *Megamonas*, and *Faecalibacterium* were significantly depleted (Figure S1G). In the young group, members of *Faecalibacterium*, *Megamonas*, and *Coprococcus* were enriched, while *Christensenellaceae R‐7*, *Escherichia–Shigella*, and *Klebsiella* were depleted (Figure S1H). The older group was characterized by enrichment of *Pseudomonas*, *Prevotella*, and *Enterobacter*, accompanied by a marked loss of beneficial commensals, including *Clostridium leptum*, *Parasutterella*, and *Bifidobacterium* (Figure S1I). Strikingly, centenarians exhibited a distinct microbial profile with enrichment of *Clostridium sensu stricto 1*, *Epulopiscium*, *Ruminococcaceae*, *Akkermansia muciniphila*, *Pseudomonas*, and *Clostridium scindens*, while potentially beneficial taxa such as *Bacteroides plebeius*, *Faecalibacterium*, and *Butyricicoccus* were significantly reduced (Figure S1J). Further pair‐wise comparisons showed that 57 ASVs were significantly enriched and 25 ASVs were depleted in the older group compared to young group, while 4081 ASVs remained unchanged. In the older group, the top 10 ASVs that increased in abundance were represented by *Pseudomonas*, *Klebsiella*, *Lactobacillus mucosae*, *Coprococcus*, and *Escherichia–Shigella*, while the depleted ASVs dominated by members of *Faecalibacterium*, *Bifidobacterium* (such as *B. adolescentis* and *B. longum*), and *Ruminococcaceae* (Figure 1B). Compared with older group, centenarians exhibited 50 ASVs enriched and 124 ASVs depleted accompanied by 5870 ASVs unchanged. Notably, enriched ASVs in centenarians included *Clostridium sensu stricto 1*, *Anaerotruncus colihominis*, *Ruminococcaceae UBA1819*, *Eggerthella*, *Epulopiscium*, *Clostridium innocuum*, *Bifidobacterium*, and *Clostridium scindens* (Figure 1C).

We next identified microbial keystone taxa by constructing co‐occurrence patterns coupled with microbial networks in the three age groups. No significant differences were observed by network layouts in the node and edge counts among them (young: 323 nodes, 1325 edges; older: 367 nodes, 1381 edges; centenarian: 372 nodes, 1392 edges; Figure 1D–F). Network topological analysis further revealed that centenarians exhibited more stable and integrate microbial network that was assembled with young group, compared with older group (Figure S2). Subsequently, keystone taxa characterized by high degree and closeness centrality but low betweenness centrality were identified as potential indicators of these network‐level alterations in centenarians (Table S2). A subset of meaningful keystone taxa representing both network hubs and differential abundance were derived by intersecting putatively enriched ASVs (Figure 1G–L). Specifically, 22, 18, and 13 keystone taxa were identified in corresponding age groups. Enriched ASVs, including *Ruminococcaceae* and *Holdemania* were also highlighted as meaningful keystone taxa in the young group (Figure 1H). In the older group, mainly enriched ASVs including *Lachnospiraceae*, *Pseudomonas*, *Clostridia* and *Rhodococcus* can be regarded as keystone taxa (Figure 1J). Notably, *Clostridium aldenense* (*C. aldenense*) and *Clostridium scindens* (*C. scindens*) were identified as both significantly enriched ASVs and meaningful keystone taxa in centenarians (Figure 1L). Taken together, these results suggest that centenarians harbor distinctive microbial keystone taxa dominated by members of genus *Clostridium*.

### Identification of metabolic signatures in centenarians

**Figure 2:**
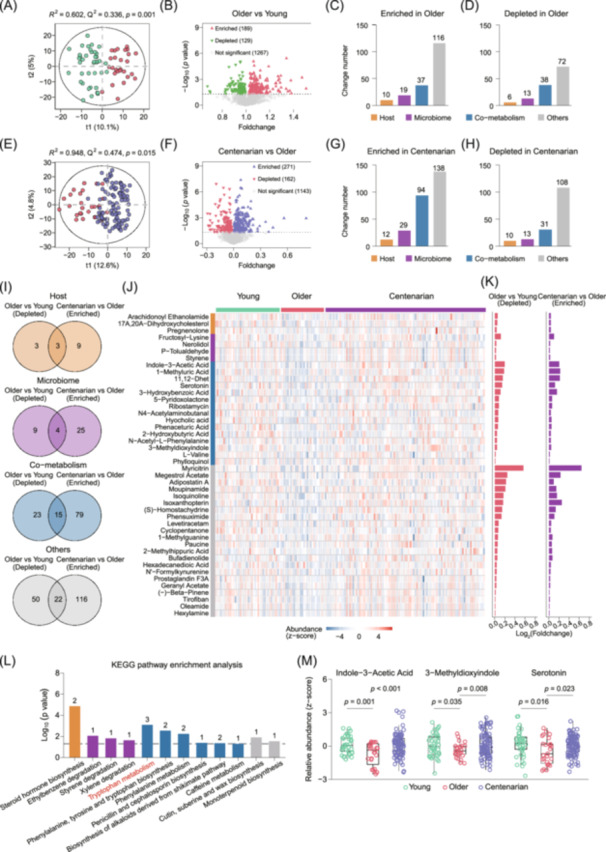
Metabolic signatures associated with the gut microbiota in centenarian. (A) Orthogonal partial least squares discriminant analysis (OPLS‐DA) of untargeted metabolomics profiles. Colors indicates the young (green) and older (red) group. The axes represent the first two components (t1 and t2), with *R*² and *Q*² values and the *p*‐values. (B) Differential metabolites between the older and young groups. Metabolites with FoldChange > 0 and −Log_10_ (*p*‐value) > 1.3 were defined as significantly altered metabolites. (C, D) Distribution of enriched and depleted metabolites, categoried as host‐derived (orange), microbiome‐derived (purple), co‐metabolized (blue), or others (gray). (E–H) Same analysis as in (A–D), comparing centenarian and older groups. (I) Overlapping metabolites that are depleted in the older versus young groups and enriched in the centenarian versus older groups, categorized as host‐derived, microbiome‐derived, co‐metabolized, or others. (J) Heatmap showing the relative abundance of overlapping metabolites. Each column represents a sample, and each row represents a metabolite; color intensity indicates abundance (*z*‐score normalization). (K) Fold changes of overlapping metabolites between groups. Red bars (left) represent metabolites depleted in the older group relative to the young group, while purple bars (left) indicate metabolites enriched in the centenarian group relative to the older group. (L) Kyoto Encyclopedia of Genes and Genomes (KEGG) pathway enrichment analysis of overlapping metabolites. Bar colors represent pathways associated with host (orange), microbiome (purple), co‐metabolism (blue), and other (gray) categories. Numbers above the bars indicate the number of metabolites mapped to each pathway. (M) Relative abundance changes of metabolites involved in tryptophan metabolism, including indole‐3‐acetic acid, 3‐methyldioxyindole, serotonin. Statistical significance was determined using two‐tailed Student's *t*‐test (two‐group comparisons). Significance indicated by asterisks between groups (*p < 0.05, **p < 0.01, ***p < 0.001).

As microbial reconfiguration and network attribution reshape host metabolome during aging, we next used untargeted HPLC‐MS‐based metabolomics to screen host metabolic profiling in fecal samples of human individuals with age. Global annotation revealed substantial metabolites derived from host (*N* = 88), microbiome (*N* = 132), co‐metabolism (*N* = 438), and others (*N* = 920) (Figure S3A). Multivariate partial least squares discriminant analysis (PLS‐DA) coupled with permutation‐based dissimilarity testing suggested significant separations between the paired age groups (older vs. young: *p* = 0.003; centenarian vs. older: *p* = 0.012) (Figure S3B,C). Similar to the featured microbial ASVs, featured metabolites can be defined as metabolic signatures that were involved in multiple metabolic pathways such as phenylpropanoid biosynthesis, amino acid metabolism, and steroid biosynthesis (Figure S3D–H). Multivariate pairwise comparison analyses showed a marked separation of fecal metabolome between young and older groups (*R*² = 0.602, *Q*² = 0.336, *p* = 0.001; Figure 2A). Compared with young group, relatively older adults exhibited significantly enriched 189 metabolites with 10 host‐derived, 19 microbiome‐derived, and 37 co‐metabolites (Figure 2B,C), whereas reduced 129 metabolites with six host metabolites, 13 microbial metabolites, and 38 co‐metabolites (Figure 2D). Compared with older group, centenarians exhibited markedly differential metabolome (*R*² = 0.948, *Q*² = 0.474, *p* = 0.015; Figure 2E), manifested by significantly upregulated 271 metabolites (12 host metabolites, 29 microbial metabolites, and 94 co‐metabolites) and 162 downregulated metabolites (29 host metabolites, 92 microbial metabolites, and 10 co‐metabolites) (Figure 2F–H).

Next, a Venn diagram for intersection analysis between the paired groups was performed to specifically identify metabolic signatures in centenarians. The results showed that the levels of three host metabolites (arachidonoyl ethanolamide, 17 A,20A‐dihydroxycholesterol, and pregnenolone), four microbial metabolites (fructosyl‐lysine, nerolidol, p‐tolualdehyde, and styrene), and 15 co‐metabolites of host and bacteria (e.g., indole‐3‐acetic acid, 1‐methyluric acid, and serotonin) were markedly enriched in centenarians and simultaneously depleted in older group (Figure 2I–K). Further pathway enrichment analysis of Kyoto Encyclopedia of Genes and Genomes (KEGG) revealed that tryptophan metabolism was the most significantly enriched co‐metabolic pathway in human feces during aging (Figure 2L). Notably, centenarians exhibited higher levels of indole‐3‐acetic acid (IAA), 3‐methyldioxindole, and serotonin than those in feces of older individuals (Figure 2M). Collectively, these results suggest that age‐related featured metabolites were progressively changed and tryptophan metabolites, especially endogenous IAA, may be potential metabolic signatures of centenarians.

### Tryptophan metabolism is linked to microbial keystone taxa in centenarians

**Figure 3:**
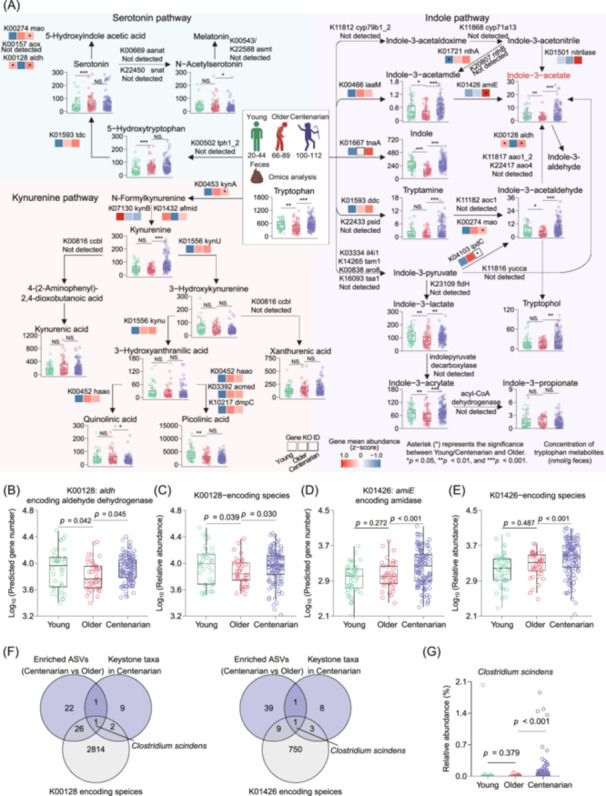
Correlation between the function potential of the gut microbiome and tryptophan metabolism. (A) Integrated analysis of gut microbial gene abundance and metabolite concentrations involved in the serotonin, kynurenine, and indole pathways of tryptophan metabolism. Box plots with dots show the absolute concentrations of metabolites in the young, older, and centenarian groups. Statistically significant differences between groups are indicated by asterisks (*p < 0.05, **p < 0.01, ***p < 0.001). Heatmaps above the arrows indicate the relative abundance of microbial genes involved in the corresponding metabolic steps. Color intensity indicates the mean abundance (*z*‐score normalization) of each group. Statistically significant differences between the young and centenarian groups compared to the older group are indicated by asterisks. (B) Predicted gene number of microbiome‐derived *aldh*. (C) Relative abundance of *aldh*‐encoding species. (D) Predicted gene number of microbiome‐derived *amiE*. (E) Relative abundance of *amiE*‐encoding species. (F) Venn diagram showing the overlap among enriched ASVs (centenarian vs. older groups), keystone taxa in centenarians, and species encoding K00128 or K014026. (G) Relative abundance of *C. scindens*. Statistical significance was determined using two‐tailed Student's *t*‐test (two‐group comparisons). Significance indicated by asterisks between groups (*p < 0.05, **p < 0.01, ***p < 0.001).

Given that tryptophan metabolism is one of the critical pathways in centenarians, HPLC‐QQQ‐MS‐based targeted metabolomics was employed to quantitatively measure the concentration of fecal metabolites involved in tryptophan metabolism in human with age (Figure 3A). In the gastrointestinal tract, microbiota‐derived tryptophan metabolism yields potent bioactive metabolites involved in three major metabolic pathways, one of which is the direct transformation of tryptophan by the gut microbiota into indole and its derivatives. Targeted quantification showed that centenarians exhibited significantly higher levels of fecal indole metabolites, including indole‐3‐acetamide (IAM), indole, tryptamine, IAA, indole‐3‐acetaldehyde (IALD), indole‐3‐lactate (ILA), and indole‐3‐acrylate in feces than relatively older group (Figure 3A). Accordingly, functional prediction using PICRUSt2 revealed that amidase (K01426 *amiE*) and aldehyde dehydrogenase (K00128 *aldh*), enzymes encoding the conversion of IAM and IALD into IAA, were markedly enriched in centenarians (Figure 3B,D). Bacterial species encoding K00128 *aldh* and K01426 *amiE* were also predicted to be enriched in centenarians (Figure 3C,E). Specifically, Venn diagrams indicated that *C. scindens* contributing to both K00128 *aldh* and K01426 *amiE* was commonly enriched in centenarians with microbial ASVs and keystone taxa (Figure 3F,G). The genes encode K00128 *aldh* and K01426 *amiE* that catalyze the biosynthesis step for IAA, suggesting a possible correlation between *C. scindens* and IAA production. In summary, these findings reveal that microbial keystone taxa *C. scindens* and indole metabolites, especially IAA, represent typical microbiome–metabolite–host associations in centenarians.

### *C. scindens* produces IAA by bacterial enzymes ALDH and AMIE

**Figure 4:**
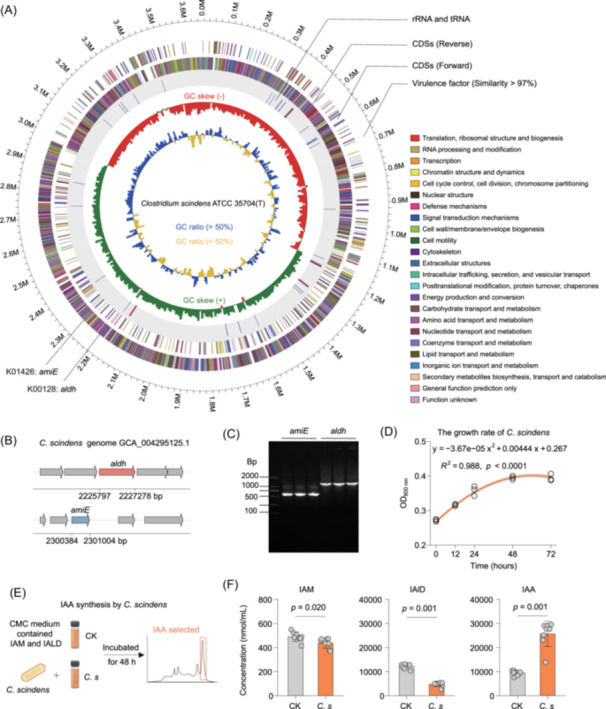
Genomic and functional characterization of *C. scindens* and *in vitro* validation of indole‐3‐acetic acid (IAA) production. (A) Circular genome map of *C. scindens* ATCC 35704(T). Tracks from the innermost to outermost circles represent GC skew, GC ratio, GC content, rRNA and tRNA genes, coding sequences (CDSs), and virulence factors. Colors represent various functional categories. Genes encoding amidase (K01426: *amiE*) and aldehyde dehydrogenase (K00128: *aldh*) are highlighted. (B) Genomic loci of *amiE* (red) and *aldh* (blue) in the *C. scindens* genome. (C) PCR amplification products of *amiE* (left) and *aldh* (right) in *C. scindens*. (D) Growth curve of *C. scindens* over 72 h. The curve was fitted using a quadratic regression model. (E) Schematic overview of the experimental design for validating IAA production. (F) Absolute concentrations of indole‐3‐acetamide (IAM), indole‐3‐acetaldehyde (IALD), and IAA after 48 h of *C. scindens* incubation. Statistical significance was determined using two‐tailed Student's *t*‐test (two‐group comparisons).

To identify whether or how IAA is produced by *C. scindens*, we analyzed the whole‐genome sequencing of *C. scindens* using functional annotation. The assembled genome of *C. scindens* ATCC 35704 (T) comprises a circular chromosome of 3658040 bp and a GC content of 46.2% (Figure 4A). A total of 12 rRNA and 57 tRNA genes coupled with clear GC skew patterns and wide functional genes (CDSs) distribution were identified across both forward and reverse strands. Meanwhile, amino acid, carbohydrate, and lipid metabolism, as well as secondary metabolite biosynthesis and transport were dominant and underscore its metabolic versatility in the gut microecosystem. Notably, the absence of virulence‐associated genes (>97% similarity) in the *C. scindens* genome suggested its consideration as a safe candidate strain for host (Figure 4A). Further genomic analysis identified the presence of genes encoding amidase (K01426 *amiE*, 2225797–2227278 bp) and aldehyde dehydrogenase (K00128 *aldh*, 2300384–2301004 bp) within the *C. scindens* genome (Figure 4B) that were further confirmed by PCR amplification of *amiE* and *aldh* encoding sequences in *C. scindens* strain (Figure 4C), providing genomic evidence for its capacity to produce IAA. This notion was supported by subsequent in vitro experimental results, which showed that *C. scindens* can successfully convert the precursors IAM and IALD into IAA (Figure 4D–F). Specifically, quantitative analyses showed significant decreases in concentrations of IAM and IALD from 490.55 and 12155.55 nmol/mL to 436.65 and 4701.42 nmol/mL, respectively, whereas the IAA level was increased from 9470.30 to 25658.61 nmol/mL under the condition of *C. scindens* culture (Figure 4F). These results indicate that keystone taxa *C. scindens* in centenarians produce IAA from its substrates by bacterial ALDH and AMIE. Especially, *C. scindens*‐derived ALDH‐mediated conversion of IALD might be the predominant biosynthesis of IAA.

### *C. scindens* mitigates intestinal aging and enhances microbial network

**Figure 5:**
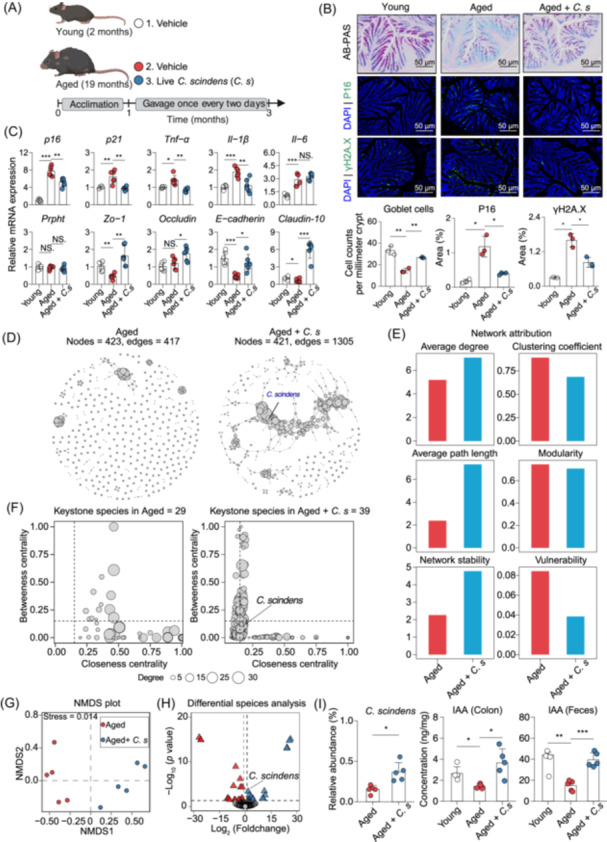
Supplementation with *C. scindens* mitigates intestinal aging. (A) Experimental design for *C. scindens* supplementation in aged mice. (B) Representative images of Alcian blue‐periodic acid‐Schiff (AB‐PAS) staining and immuno‐staining for P16 and γH2AX, with quantitative analysis (*n* = 3). Scale bar, 50 μm. (C) Relative mRNA expression levels of *p16*, *p21*, *Tnf‐α*, *Il‐1β*, *Il‐6*, *Ptprh*, *Zo‐1*, *Occludin*, *E‐cadherin*, and *Claudin*‐*10* (*Cldn10*) genes in colon tissues (*n* = 6). (D) Effect of *C. scindens* supplementation on the co‐occurrence network of gut bacterial community in aged mice. Node size represents the degree of species in network. Only robust correlations in which |*r*| (Pearson's correlation coefficient) was >0.75 and *p* (Pearson significance coefficient) was <0.01 were visualized. (E) Changes in the topological attributions of co‐occurrence network after *C. scindens* supplementation. (F) Changes in the numbers of keystone species after *C. scindens* supplementation. (G) Effect of *C. scindens* supplementation on the dissimilarity in gut microbiome based on Bray‐Curtis distance. The stress value of non‐metric multidimensional scaling (NMDS) analysis is shown on left top of plot. (H) Differential species between without and with *C. scindens* supplementation groups. The species with Log_2_|Foldchange| > 1 and −Log_10_ (*p*‐value) > 1.3 were selected as different species. Red triangle represents the species enriched in the gut of aged mice, while blue triangle represents the species enriched in the gut of young mice, respectively. Unchanged species were represented by gray dots. (I) Relative abundances of *C. scindens* and the concentrations of IAA in colon tissue and fecal samples. Statistical significance was determined using two‐tailed Student's *t*‐test (two‐group comparisons). Significance indicated by asterisks between groups (*p < 0.05, **p < 0.01, ***p < 0.001).

To evaluate whether *C. scindens* colonization can ameliorate intestinal aging, aged mice (19 months) following 1 month‐adaptation were orally administered with live *C. scindens* (or vehicle control) twice per week for 2 months (Figure 5A). The Alcian blue‐periodic acid‐Schiff staining (AB‐PAS) analyses (Figure 5B) of colonic tissues showed that supplementation with live *C. scindens* markedly restored colonic structure, manifested by significant upregulation of the number of goblet cells per crypt that were markedly reduced in aged mice (Figure 5B). Immunofluorescence staining with P16 and γH2A histone family member X (γH2AX), well‐known markers related to cell senescence and DNA damage, suggested that *C. scindens* supplementation significantly mitigated cell senescence and DNA damage in colonic tissues of aged mice (Figure 5B). Concurrently, RT‐qPCR analysis indicated that *C. scindens* supplementation strikingly downregulated mRNA levels of typical cell senescence markers (*p16* and *p21*) and restored key intestinal barrier–associated genes (*Zo‐1*, *Occludin*, *E‐cadherin*, and *Claudin‐10* (*Cldn10*)) (Figure 5C). Moreover, supplementation of *C. scindens* significantly downregulated the levels of pro‐inflammatory cytokines such as *Tnf‐α*, *Il‐1β*, and *Il‐6* in colon tissues of aged mice (Figure 5C).

It is worth noting that *C. scindens* supplementation markedly enhanced the microbial network connectivity, as shown by increased edges from 417 to 1305 (Figure 5D), which was supported by the significantly increased average degree, path length and network stability together with decreased vulnerability (Figure 5E). Further analysis revealed that *C. scindens* supplementation markedly increased the number of keystone taxa from 29 to 39 in gut microbiota of aged mice, leading to *C. scindens* as a keystone node (Figure 5F and Table S3). NMDS analysis showed a slight separation between pre‐ and post‐treatments of *C. scindens* with moderate microbiota compositional shift (Stress = 0.104) (Figure 5G), which was confirmed by volcano analysis showing only 15 enriched and 17 depleted species (7.37% of total species) (Figure 5H). In addition, *C. scindens* successfully colonized in the gut and significantly elevated levels of IAA in both colon and feces of aged mice (Figure 5I). Taken together, these results indicate that *C. scindens* is capable of mitigating intestinal aging and enhancing the gut microecology to maintain intestinal homeostasis.

### *C. scindens*‐derived IAA mitigates intestinal aging via AHR‐dependent signaling

**Figure 6:**
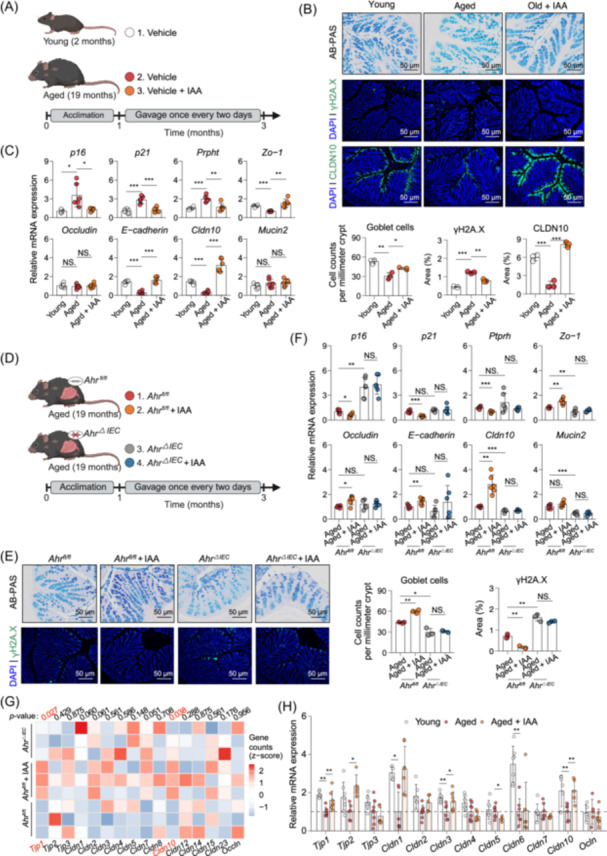
IAA supplementation mitigates intestinal aging and promotes CLDN10 expression in vivo via intestinal aryl hydrocarbon receptor (AHR) signaling. (A) Experimental design for IAA supplementation in aged mice. (B) Relative mRNA expression levels of *p16*, *p21*, *Ptprh*, *Zo‐1*, *Occludin*, *E‐cadherin*, *Cldn10*, and *Mucin2* genes in colon tissues (*n* = 6). (C) Representative images of AB‐PAS staining and immuno‐staining of γH2AX and CLDN10, with corresponding quantification (*n* = 3). Scale bar, 50 μm. (D) Experimental design for IAA supplementation in aged mice with intestinal *Ahr* knockout. (E) Same analysis as in (B) for IAA supplementation in aged mice with intestinal *Ahr* knockout. (F) Same analysis as in (C) for IAA supplementation in aged mice with intestinal *Ahr* knockout. (G) Transcriptomic analysis showing the effect of IAA supplementation on intestinal barrier‐associated gene expression in the colon of aged mice with intestinal *Ahr* knockout. (H) Effect of IAA supplementation on the mRNA levels of intestinal barrier‐associated genes in colon of aged mice. Statistical significance was determined using two‐tailed Student's *t*‐test (two‐group comparisons). Significance indicated by asterisks between groups (*p < 0.05, **p < 0.01, ***p < 0.001).

Given that *C. scindens* producing IAA is able to mitigate intestinal aging, we next evaluated whether and how IAA can exert anti‐aging effects on intestinal decline. Aged mice (19 months old) following 1 month‐adaptation were orally administered by gavage with IAA (50 mg/kg body weight) once every 2 days for 2 months (Figure 6A). Similar to *C. scindens* supplementation, IAA exposure markedly improved intestinal pathophysiological status and histological structure of aged mice. AB‐PAS staining showed that IAA supplementation restored mucosal barrier integrity with increased goblet cell numbers, which were significantly reduced in aged mice (Figure 6B). CLDN10 and γH2AX immunofluorescence staining indicated that IAA treatment significantly reduced DNA damage and restored gut barrier of aged mice (Figure 6B). Meanwhile, IAA supplementation significantly downregulated mRNA levels of cell senescence markers (*p16* and *p21*) and restored the expression of several key intestinal barrier–associated genes, such as *Zo‐1*, *E‐cadherin*, and *Cldn10* (Figure 6C). Since IAA is known as an endogenous ligand of AHR, we subsequently used intestinal epithelial cell‐specific *Ahr* knockout (*Ahr* ^ΔIEC^) mice upon IAA supplementation to verify AHR‐mediated mechanism (Figure 6D). Expectedly, IAA supplementation did not exhibit significant improvement in intestinal aging phenotypes of aged *Ahr* ^ΔIEC^ mice, shown with no significant restoration in intestinal morphology and goblet cell numbers (Figure 6E). Notably, IAA‐treated aged *Ahr* ^ΔIEC^ mice exhibited similar intestinal epithelial DNA damage to aged *Ahr* ^ΔIEC^ mice, manifested by no significant changes in γH2AX immunofluorescence area percentage (Figure 6E), mRNA levels of cell senescence markers (*p16* and *p21*) and gut barrier function–associated genes (*Zo‐1*, *Occludin*, *E‐cadherin*, *Cldn10* and *Mucin2*) (Figure 6F). Transcriptomic profiling (RNA‐seq) (Figure 6G) coupled with RT‐qPCR analysis (Figure 6H) showed that IAA supplementation markedly restored mRNA levels of intestinal barrier–associated genes, especially *Tjp1* and *Cldn10*, which were significantly reduced in the colon of aged mice and aged *Ahr* ^ΔIEC^ mice.

**Figure 7:**
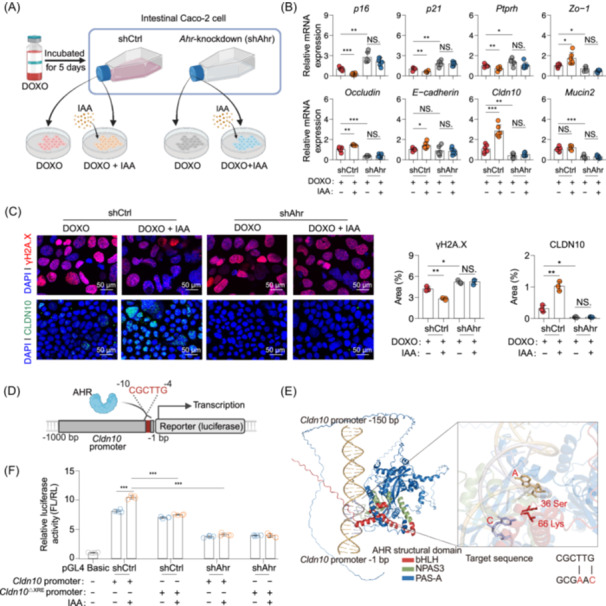
IAA supplementation attenuates cellular senescence in vitro via intestinal AHR‐CLDN10 signaling. (A) Schematic workflow of IAA treatment in senescent wild‐type (WT) and *Ahr*‐knockdown (shAhr) Caco‐2 cells. (B) Relative mRNA expression levels of *p16*, *p21*, *Ptprh*, *Zo‐1*, *Occludin*, *E‐cadherin*, *Cldn10*, and *Mucin2* genes in colon tissues (*n* = 6). (C) Representative immuno‐staining images and corresponding quantification of γH2AX and CLDN10 (*n* = 3). Scale bar, 50 μm. (D) Schematic of the *Cldn10* promoter region and its transcription activation by AHR. The AHR‐binding motif (CGCTTG) is indicated, with transcription directed toward the luciferase reporter gene. (E) Molecular dynamics simulation of AHR binding to the *Cldn10* promoter. The bHLH, NPAS3, and PAS‐A domains of AHR are shown in red, blue, and green, respectively. Key residues (Ser36, Lys66) involved in interaction with the target sequence are highlighted. (F) Relative luciferase activity of the *Cldn10* promoter in response to AHR activation. Statistical significance was determined using two‐tailed Student's *t*‐test (two‐group comparisons). Significance is indicated by asterisks between groups (*p < 0.05, **p < 0.01, ***p < 0.001).

To further investigate the potential mechanism of IAA‐mitigating intestinal aging, we successfully established a cell senescent model using Caco2 cells with and without *Ahr*‐knockdown (shAhr) treated with doxorubicin (DOXO, 250 nm) and IAA (50 μM) for 7 days (Figure 7A). CLDN10 and γH2AX immunofluorescence staining suggested that senescent wild type (WT) Caco2 cells by DOXO exhibited marked DNA damage and gut barrier disruption, both of which were significantly restored following IAA treatment (Figure 7B). These observations were further confirmed by quantitative analyses showing a significant decrease in γH2AX‐positive area and increased CLDN10 levels of aged cells (Figure 7B). Notably, RT‐qPCR analysis suggested that IAA treatment induced markedly transcriptional restoration of senescence markers (*p16* and *p21*) and gut barrier‐associated genes (*Zo‐1*, *Occludin*, *E‐Cadherin*, and *Cldn10*) in senescent cells (Figure 7C). Nevertheless, no significant restorations in DNA damage and related cellular senescence phenotypes were observed in DOXO‐induced senescent cells with shAhr upon IAA supplementation (Figure 7B,C). These in vivo and in vitro results reveal that IAA exhibits marked intestinal AHR‐dependent alleviation effects on aging phenotypes such as gut barrier dysfunction and DNA damage of both aged mice and senescent cells.

### Activation of AHR by IAA mitigates CLDN10‐related gut barrier dysfunction

Of particular note, a putative AHR response element (XRE) as one binding site of *Cldn10* promoter from −10 to −4 bp was found to interact with the core motif CGCTTG of AHR protein via simulation of the JASPAR database (Figure 7D). Subsequently, we used molecular dynamics (MD) simulations to theoretically predict the possible interplay between AHR and DNA sequence of *Cldn10* promoter (Figure 7E). The results showed that AHR complex forms a bHLH‐PAS heterodimer with NPAS3 and binds to the target DNA sequence of *Cldn10* promoter via its basic helix‐loop‐helix (bHLH) and PAS‐A domains (Figure 7E). As shown in the model, the binding site was mapped to a conserved XRE motif (5'‐GCGAAC‐3'), which is the typical AHR response element. Notably, structural analysis highlighted a potential base‐specific interaction between residues Ser36 and Lys66 of bHLH and the target DNA sequence (CGCTTG/GCGAAC) of *Cldn10* promoter (Figure 7E), suggesting direct base recognition at the binding site. A pGL4‐Basic *Cldn10*‐promoter‐luc plasmid spanning from −10 to −4 bp of *Cldn10* promoter cloned upstream of luciferase was constructed in HEK 293T cells to experimentally verify AHR binding to *Cldn10* promoter (Figure 7F). After transfection of *Cldn10* luciferase reporter vectors into HEK 293T cells with and without AHR knockdown (shAhr), luciferase reporter assays indicated that IAA treatment markedly enhanced luciferase activity of *Cldn10* reporter, whereas no significant changes were observed in HEK293T cells with both XRE mutation and *Ahr*‐knockdown upon IAA administration (Figure 7F). These experimental results, coupled with theoretical simulation, provide structural evidence that activation of AHR by IAA mitigates aging‐related gut barrier dysfunction via binding to *Cldn10* promoter.

## DISCUSSION

The gut microbiota, an essential regulator of pathophysiological processes, the immune system, and host metabolism, profoundly changes during aging [10, 11, 17]. Centenarians exhibit a unique pattern of the gut microbiome, characterized by increased microbial community evenness, enrichment of beneficial commensals (e.g., *Bacteroidetes*), and depletion of potential pathogenic bacteria, which are similar to young individuals and may contribute to longevity [16, 17, 27]. However, less work has been done on microbial interaction network within the complex microecology, in which keystone taxa are dominant for microbial community composition and function irrespective of their abundance [18, 33]. In this study, we present a landscape of aging‐related patterns of the gut microbiome and metabolic signatures, especially microbial keystone taxa and signatures of centenarians, by employing co‐occurrence patterns coupled with microbial networks. Specifically, centenarians harbor distinctive keystone taxa dominated by members of *Clostridium*, of which *C. scindens* produces IAA production from tryptophan metabolism, probably contributing to longevity and reduced susceptibility to age‐related diseases.

Gut microbes engage in symbiosis, competition, and cross‐feeding (metabolic hand‐offs), collectively shaping microbial ecological community [34, 35]. Nevertheless, microbe–microbe interactions forming microbial networks, one of the key factors for gut homeostasis, has been markedly underexplored. Here, age‐related gut microbial characteristics showed a marked depletion of beneficial keystone taxa such as *Faecalibacterium*, *Bifidobacterium* (e.g., *B. adolescentis* and *B. longum*), and members of *Ruminococcaceae* family together with an enrichment of opportunistic bacteria such as *Pseudomonas*, *Klebsiella*, and *Escherichia–Shigella* observed in the older group. These opportunistic taxa have been shown to link to increased susceptibility to systemic inflammation in the elderly [36], suggesting that the age‐related microbiome shifts may contribute to aging and aging‐related inflammation and frailty [22, 37]. Centenarians exhibited markedly enriched abundance of ASVs, including *Bifidobacterium*, *Anaerotruncus colihominis*, *Ruminococcaceae UBA1819*, *Eggerthella*, and *Epulopiscium*, which were consistent with previous abundance‐based results of cross‐sectional and longitudinal studies in Chinese and Japanese cohorts [16]. Contrary to what we currently observed, a recent study reported that abundant *Bacteroidetes* in the gut microbiome was a potential beneficial bacterium and a hallmark in centenarians [17]. In this study, our network analysis did not identify *Bacteroidetes* as keystone taxa in centenarians, although *Bacteroidetes* are often reported as a hallmark of centenarian microbiota in terms of its relative abundance [38]. In addition, the members of *Clostridium* genus (*C. clostridioforme* and *C. innocuum*), have been previously associated with increased frailty severity in older adults [39]. Instead, our co‐occurrence network highlights a different set of *Clostridium* taxa that exhibit higher degree and closeness centrality and low betweenness centrality in centenarians. Despite not being the most differentially abundant biomarker (0.102%) in centenarians, *C. scindens* is known to promote immunomodulation and maintain metabolic homeostasis [40, 41]. Notably, *C. scindens* supplementation significantly enhanced the microbial network connectivity or stability and restoring intestinal homeostasis in aged mice. This contrast highlights a novel insight that *Bacteroidetes* may be abundantly dominant in centenarians but they may not necessarily occupy the most influential functional niches within the microbial interaction network. In addition, *C. scindens*, as a keystone taxon, serves as a critical regulator in microbe‐host interplay leading to IAA production, both of which may contribute to longevity in centenarians.

A novel mechanistic link revealed by in vivo and in vitro experiments is that production of IAA by *C. scindens* via bacteria enzymes AMIE and ALDH may alleviate gut barrier dysfunction and intestinal aging in aged mice. Similarly, previous studies demonstrated that *C. scindens* can inhibit the growth and infection of the gram‐positive pathogen *C. difficile* both in vitro and in mice, thus maintaining gut homeostasis by promoting isoallolithocholic acid (iso‐LCA) production in centenarians [42]. It is of particular note that other members of the gut microbiota, including *Lactobacillus*, can also produce IAA to alleviate intestinal aging by repairing DNA to maintain genome stability, which further suggests the complexity of microbe–host interactions [43]. Mechanistically, *C. scindens*‐derived IAA markedly restored intestinal function decline by mitigating aging via AHR‐mediated intestinal CLDN10, one of the typical functional tight junction proteins in mammals. AHR is a promiscuous receptor that can be activated by a spectrum of microbially derived tryptophan metabolites (including multiple indole derivatives), and centenarians exhibit markedly increased levels of multiple indole derivatives. It is conceivable that other enriched indoles may also contribute to the AHR‐dependent effects of gut barrier function, potentially in a synergistic manner. Here, IAA is identified as the most significantly enriched indole derivative, suggesting that it acts as a potent AHR ligand to restore the epithelial barrier by directly targeting *Cldn10* promoter. Although several studies have shown that IAA and its derivative 5‐hydroxyindole‐3‐acetic acid (5HIAA) upregulate the expression of *Claudin* and *Occludin* genes to counter intestinal inflammation via AHR signaling [44, 45], the mechanism underlying the interaction between AHR and these genes remains unclear. Here, we provide solid evidence that IAA supplementation promoted direct regulation of *Cldn10* expression by facilitating AHR binding to its promoter region. Structurally, bHLH and PAS‐A domains of AHR upon IAA combined with DNA sequence of *Cldn10* promoter, forming a stable complex that enhanced *Cldn10* gene transcriptional activity.

These findings highlight the dual role of keystone taxon *C. scindens*, which served not only as microecological stabilizers maintaining microbial stability through the microbe–microbe interactions, but also as functional mediators that directly promote host homeostasis via microbe–host interplay. Although we identified keystone taxon *C. scindens* and metabolic signature IAA contributing to intestinal homeostasis in centenarians, other microbial keystone taxa and their metabolites, such as SCFAs, bile acids, and indole derivatives, may also mediate microbial community and host homeostasis via microbiota–host crosstalk, which warrants further investigations. Moreover, our correlation‐based networks primarily serve as a hypothesis‐generating tool and do not definitively reflect direct ecological interactions. The cross‐sectional human data identify correlations rather than direct causality for human longevity. Comprehensive metadata for all possible confounders were also not fully available for formal sensitivity or stratified analyses due to the nature of this retrospective cohort. It is essential for expanding to longitudinal populations with replication by integrating causal intervention methods [46] and deep learning frameworks [47] to better identify keystone taxa.

## CONCLUSION

In this study, we identify microbial keystone taxon *C. scindens* and its metabolite IAA as a metabolic signature in centenarians using a co‐occurrence pattern approach and microbial networks. Our results demonstrate that *C. scindens* regulates the synthesis of IAA through its own enzymes AMIE and ALDH. We reveal that both *C. scindens* and IAA play crucial roles in promoting gut microecological stability and host homeostasis via microbe–microbe and microbe–host interactions. These findings provide a mechanistic basis for targeting keystone taxa and microbe–host interactions in mitigating aging and aging‐related disorders.

## METHODS

### Ethics statement and human cohort study

The study protocol was reviewed and approved by the Ethics Committee of AIage Life Science Corporation (approval no. AS‐LL‐ZD‐001) and the Ethics Committee of the First Affiliated Hospital of Guangxi Medical University (approval no. 2020‐KT‐050). All procedures involving human participants were conducted in accordance with the Declaration of Helsinki. Written informed consent was obtained from all participants prior to the collection of fecal samples. Fecal samples from healthy individuals were collected and classified into three groups: young (20–44 years old, *n* = 46), older (66–89 years old, *n* = 39), and centenarian (100–112 years old, *n* = 113). All participants were required to fast for 12 h prior to sampling. The fecal samples were subsequently subjected to microbiome and metabolome analyses.

### Animal experiments

All animal procedures were authorized by the Animal Ethics Committee of the Innovation Academy for Precision Measurement Science and Technology, CAS (APM No: APM24005A, China). Male C57BL/6J mice, including young (2 months) and aged (19 months) mice as well as genetically modified strains (*Ahr* ^flox/flox^ and gut‐specific *Ahr* knockout, *Ahr* ^ΔIEC^), were obtained from licensed vendors (Wukong Biotechnology and Sai Ye Biotechnology, Jiangsu, China) and housed under specific pathogen‐free conditions. Each mouse was maintained with sterilized food and autoclaved water ad libitum under a controlled light‐dark cycle (12 h:12 h) at 22 ± 2°C and 55% ± 10% relative humidity. For experiments, all mice were co‐housed by age cohort in standard cages, and a maximum of two mice per cage were used to avoid cage effects. Body condition, food intake, and general health were monitored throughout the study.

For *C. scindens* supplementation, three groups were established following a 1‐month acclimation: young mice (Young, *n* = 6), aged mice (Aged, *n* = 6), and aged mice receiving live *C. scindens* (Aged + *C.s*, *n* = 6). For bacterial intervention, live *C. scindens* was prepared at a concentration of 1 × 10⁹ CFU/mL, and each mouse received 200 µL per gavage once every 2 days for 2 months, while the young and aged groups received an equal volume of PBS.

For IAA intervention, 19‐month‐old mice were randomly divided into two groups: aged mice (aged, *n* = 6) and aged mice supplemented with IAA (Aged + IAA, *n* = 6), in addition to the young mice (Young, *n* = 6). After a 1‐month acclimation, mice in Aged + IAA group were administered IAA dissolved in corn oil at a concentration of 50 mg/kg body weight (200 µL per gavage) once every 2 days for 2 months, while other controls received an equal volume of corn oil.

For AHR‐dependent effects of IAA intervention, 19‐month‐old *Ahr* ^flox/flox^ mice and gut‐specific *Ahr* knockout (*Ahr* ^ΔIEC^) mice (*n* = 6) were randomized into four groups: *Ahr* ^flox/flox^ + vehicle, *Ahr* ^flox/flox^ + IAA, *Ahr* ^ΔIEC^ + vehicle, and *Ahr* ^ΔIEC^ + IAA. After a 1‐month acclimation, mice assigned to IAA were administered IAA dissolved in corn oil at a concentration of 50 mg/kg body weight (200 µL per gavage) once every 2 days for 2 months, while vehicle groups received corn oil with the equal volume.

At the end of the intervention (month 3), mice were fasted overnight and euthanized. The gut tissues and fecal sample of each mouse were collected and stored at −80°C until for further analyses.

### Bacterial culture and genomic analyses

The pure strain of *C. scindens* (ATCC 35704), was obtained from the American Type Culture Collection (ATCC), originally isolated from the human gut microbiota. It was routinely cultured in PYG medium (Medium 104) supplemented with haemin (5 µg/mL) and vitamin K₁ under strictly anaerobic conditions at 37°C in a COY type B anaerobic chamber with a gas mixture of 5% H₂, 10% CO₂, and 85% N₂, with hydrogen maintained at 3.3% using an anaerobic gas infuser. The genome of *C*. scindens was annotated on EzBioCloud using a standard prokaryotic workflow. Virulence factors were screened against VFDB by BLASTP (identity ≥ 97%, coverage ≥ 80%). GC content and GC skew were calculated in sliding windows across the chromosome. Following genome annotation via the EzBioCloud database, circular genome maps were rendered and integrated multiple genomic features, including forward/reverse CDSs, rRNA/tRNA loci, KEGG/COG functional categories, virulence‐associated genes, and GC content/skew profiles. Notably, KEGG KOs corresponding to key enzymes were recovered, including *amiE* (K01426) and *aldh* (K00128). The *amiE* (K01426) and *aldh* (K00128) genes of *C. scindens* were amplified by PCR using specific primers (Table S4), respectively, and the resulting products were verified by 1% agarose gel electrophoresis.

### Caco‐2 cell culture and IAA treatment

Caco‐2 (RRID: CVCL_0025; CCTCC #GDC0153) and HEK293T (RRID: CVCL_0063; CCTCC #GDC0187) and cell lines were obtained in 2021 from the China Center for Type Culture Collection (CCTCC, Wuhan University, Wuhan, China). Both lines were routinely screened for mycoplasma and confirmed negative. HEK293T cells were cultured in Dulbecco's modified Eagle's medium (DMEM; Gibco, USA) supplemented with 10% fetal bovine serum (FBS) and 1% penicillin‐streptomycin. Caco‐2 cells were maintained in Minimum Essential Medium (MEM; Gibco) supplemented with 20% fetal bovine serum and 1% penicillin‐streptomycin at 37°C in a humidified 5% CO₂ incubator.

For *Ahr* knockdown, cells were transduced with shRNA lentiviral vectors targeting *Ahr* (shAhr; Integrated Biotech Solutions Co. Ltd., IBS). A non‐targeting shRNA lentiviral vector was used as the corresponding control (shCtrl). The shRNA target sequences were as follows: shCtrl, CCTAAGGTTAAGTCGCCCTCG; shAhr, GCTACCACATCCACTCTAAGC. Cells were transduced in complete medium containing polybrene (8 μg/mL) and, 48 h post‐infection, stably transduced cells were selected with puromycin. Knockdown efficiency and specificity were verified by immunoblotting. To generate senescent and quiescent populations in shCtrl and shAhr cells, senescence was induced with doxorubicin (DOXO, 250 nM; Sigma‐Aldrich) for 7 days; where indicated, IAA (200 μM) was added and incubated for 48 h prior to downstream assays. Quiescent controls were obtained by culturing cells in 0.2% FBS for 3 days. Cells were monitored morphologically, and the induction of quiescence and senescence was confirmed in subsequent assays.

### Dual‐luciferase reporter assay

The human *Cldn10* promoter region (−1000 to −1 bp relative to the TSS) was PCR‐amplified from genomic DNA and cloned upstream of firefly luciferase in pGL4.10 (Promega) to generate pGL4.10‐*Cldn10*‐Luc. The AHR/XRE site was disrupted by site‐directed mutagenesis (QuikChange Lightning, Agilent). HEK293T cells were seeded in 24‐well plates and, at ~70% confluence, transfected with 400 ng reporter plasmid plus 40 ng pRL‐TK Renilla control (Promega) using Lipofectamine 3000 (Invitrogen, USA). Where indicated, *Ahr*‐targeting or control shRNA plasmids were co‐transfected. After 24 h, cells were treated with vehicle or IAA (200 μM) for an additional 24 h. Firefly and Renilla activities were measured sequentially with the Dual‐Luciferase Reporter Assay System (Promega) on a GloMax 20/20 luminometer following the manufacturer's protocol. Firefly signals were normalized to Renilla for each well and expressed as relative luciferase units.

### DNA extraction

Total genomic DNA was extracted from clinically collected human fecal or mouse cecal contents samples using the Stool Genomic DNA Kit (CWBIO, cat. CW20925). DNA integrity was assessed by 1% agarose gel electrophoresis, purity was evaluated with a NanoPhotometer spectrophotometer (Implen), and concentration was quantified using the Qubit dsDNA Assay Kit on a Qubi® 2.0 Fluorometer (Thermo Fisher Scientific).

### 16S rRNA gene amplicon sequencing

To analyze the bacterial community of human cohorts, a specific region (V3–V4) of the 16S rRNA gene was amplified using the 341F/805R primer pairs (341F: 5′‐CCTACGGGNGGCWGCAG‐3′ and 805R: 5′‐GACTACHVGGGTATCTAATCC‐3′). The PCR products were purified and subsequently subjected to a second PCR to incorporate unique index sequences for sample multiplexing. The library quality was assessed using the Qubit® 2.0 Fluorometer (Thermo Fisher Scientific) and Agilent Bioanalyzer 2100 system (Agilent Technologies). The libraries were sequenced on the Illumina MiSeq platform using paired‐end 300 bp sequencing. The amplicon sequencing analysis according to the EasyAmplicon2 pipeline [48, 49]. After filtering, clean raw data were merged into the bacterial tags and further clustered into ASV with 100% sequence similarity. Based on the RDP database, the bacterial ASVs were taxonomically classified by the RDP classifier. Function potentials of ASVs were predicted using phylogenetic investigation of communities by reconstruction of unobserved states (PICRUSt2) [50].

### 16S rRNA gene full‐length sequencing

For the post‐gavage of *C. scindens*, the full‐length 16S rRNA gene of the genomic DNA from mouse cecal content was amplified using universal primers 27F (5′‐AGRGTTYGATYMTGGCTCAG‐3′) and 1492R (5′‐GYTACCTTGTTACGACTT‐3′). PCR products were gel‐purified and quality‐checked; libraries were then prepared with the PacBio SMRTbell® Prep Kit 3.0 according to the manufacturer's instructions and sequenced on a PacBio Sequel II platform. Raw reads were processed with the EasyAmplicon2 pipeline workflow: reads were demultiplexed, quality‐filtered, and clustered into ASVs at 100% sequence identity [48, 49]. Taxonomic assignment of ASVs was performed against the RDP database. To mitigate bias from unequal sequencing depth across samples, data were normalized by rarefying each sample to the minimum read count in the dataset.

### Microbiome data analysis

All analyses of bacterial community were conducted in R v4.4.2 (http://www.r-project.org). All plots were visualized using the “ggplot2” package [51]. The ASV richness and Shannon index, and NMDS metrics were calculated using the “vegan” package [52]. Analysis of similarity (ANOSIM) based on Bray‐Curtis dissimilarity was used to test the dissimilarities between groups. Differential ASVs between groups were identified using the DESeq2 package with FoldChange > 0 and Benjamini–Hochberg (BH) adjusted *p*‐value < 0.05 [53]. Featured ASVs grouped into four classes: (I) age‐related ASVs showing progressive trajectories across age groups, and (II–IV) group‐specific ASVs uniquely enriched or depleted in the young, older, or centenarian cohorts. Specifically, featured ASVs were identified and defined as the significantly altered variants in a specific group, whereas they remained unchanged in the other two groups (*p* < 0.05, determined by the Kruskal–Wallis test followed by Dunn's multiple comparisons posttest).

For co‐occurrence network, the pairwise interactions among bacterial ASVs were calculated using WGCNA package [54]. An interaction was robust when Pearson's correlation coefficient > 0.75 and BH‐adjusted *p*‐value < 0.01. The acquired adjacency matrix was generated into a co‐occurrence network using the igraph package [55]. The topological features of each network were calculated using the igraph package, including nodes, edges, average degree, clustering coefficient, average path length, and modularity. Network visualization was performed using the igraph package. To evaluate the stability of each network, the robustness index was quantified by calculating the proportion of taxa that remained when randomly removing 50% of taxa from each network [56].

Keystone taxa with the highest degree and highest closeness centrality, and the lowest betweenness centrality scores were defined from co‐occurrence networks using node‐level centrality criteria [57]. For the human cohorts, ASVs meeting all of the following were designated as keystones: degree > 9, closeness centrality > 0.235, and betweenness centrality < 0.10. For the *C. scindens* supplementation experiment, keystone taxa were ASVs with degree > 10, closeness centrality > 0.15, and betweenness centrality < 0.15. Only nodes satisfying all three thresholds within a given network were retained as keystone taxa.

### Untargeted quantification of metabolites

Untargeted metabolomics was performed on fecal sample from human cohorts as previously described [58]. Briefly, all fecal samples (10 mg) were extracted in ice‐cold methanol/acetonitrile/water (2:2:1, v/v/v) containing 0.1% formic acid and isotopically labeled internal standards, bead‐beaten, protein‐precipitated (−20°C, 30 min), cleared by centrifugation (15,000 × *g*, 4°C), pooled, vacuum‐dried, and reconstituted in 50% acetonitrile/water (0.1% formic acid). High‐resolution liquid chromatograph mass spectrometer (LC‐MS) analysis was performed with a Thermo Fisher Scientific Vanquish Horizon UHPLC System coupled with a Thermo Q Exactive HF hybrid quadrupole‐orbitrap high‐resolution mass spectrometer equipped with a heated electrospray ionization ion source as described. Each sample was analyzed in positive and negative modes with an m/z range of 150–800.

Metabolites were classified into three categories: host‐derived, microbiome‐derived, or host–microbe co‐metabolism, based on the annotation of their biosynthetic pathways in the HMDB (v5.0) and KEGG databases. Microbiome‐derived metabolites were identified as those exclusively produced by microbial enzymes (e.g., indole‐3‐acetic acid and other tryptophan derivatives). Host‐derived metabolites were defined as endogenous products of human cellular metabolism. Co‐metabolites were characterized as those resulting from biotransformation of host‐derived precursors by microbial enzymes (e.g., secondary bile acids). This classification was further validated through manual literature search to ensure biological relevance to intestinal environment.

### Targeted quantification of tryptophan metabolites

Quantification of tryptophan and its downstream metabolites was performed on fecal and gut tissue (~10 mg per sample). All samples were extracted in ice‐cold 1:1 (v/v) methanol/acetonitrile‐water containing 0.1% formic acid and spiked with d5‐tryptophan as the internal standard. Samples were homogenized on a Qiagen TissueLyser (20 Hz, 90 s), subjected to two sequential extraction/centrifugation steps, and pooled supernatants were dried under vacuum and reconstituted in 1:1 acetonitrile/water with 0.1% formic acid. LC‐MS analyses were conducted on an Agilent 1290 UHPLC coupled to an Agilent 6460 triple‐quadrupole mass spectrometer operated in multiple‐reaction‐monitoring mode (Agilent Technologies). Analyte identity was confirmed by retention‐time matching to authentic standards and characteristic qualifier/quantifier transitions; quantification used internal‐standard normalization against matrix‐matched calibration curves. Resulting concentrations were normalized to input mass for each sample.

### Metabolomic data analysis

Raw LC‐MS data were converted to mzXML format using ProteoWizard (v3.0.8789). Feature detection, retention‐time correction, and alignment were performed in XCMS (R package, v3.1.3). Metabolite annotation was based on accurate mass (mass error < 30 ppm) and MS/MS spectral matching against HMDB (https://www.hmdb.ca/), MassBank (https://massbank.jp/), LIPID MAPS (http://www.lipidmaps.org), mzCloud (https://www.mzcloud.org), and KEGG (http://www.genome.jp/kegg/). After data scaling, multivariate models were built using PLS‐DA. Model robustness and potential overfitting were evaluated by permutation testing. Discriminant metabolites were identified using a combination of variable importance in projection from PLS‐DA, Foldchange (FC), and BH *p*‐values. Finally, metabolites were categorized into host‐derived, host–microbe co‐metabolism, microbe‐derived, and other/undetermined classes. According to featured ASVs, featured metabolites were further filtered by the Kruskal–Wallis test followed by Dunn's multiple comparisons posttest, and subsequently classified as uniquely enriched or depleted in the age‐related, young, older, or centenarian cohorts.

### RT‐qPCR analysis

Total RNA was extracted from snap‐frozen colon tissues or cultured cells using TRIzol reagent (Invitrogen). First‐strand cDNA was synthesized from 2 µg of RNA with 5× All‐in‐One RT MasterMix. Quantitative PCR was performed with gene‐specific primers listed in Table S5. Relative mRNA abundance was calculated by the 2^−ΔΔCt^ method after normalization to *Gapdh* gene, and results are reported as fold change relative to the control group.

### Transcriptome sequencing (RNA‐seq) analysis

Total RNA was extracted from colon tissue, and RNA quality was confirmed with Nanodrop 2000 (ThermoFisher). Oligo dT beads were used to enrich the mRNA and cDNA library was constructed by PCR enrichment. The library was sequenced on the NovaSeq platform (Illumina). Quality controlled of the reads was performed using FastQC, and clean reads were separately mapped against the reference genome (*Mus musculus* GRCm38) with orientation mode using HISAT2 software. All clean reads were used to map into the enriched pathway from the Gene Ontology database. A heatmap was created to display the expression (*z*‐scores) of genes involved in intestinal barrier‐associated genes. The differential expression of these specific genes was analyzed using a two‐tailed Student's *t*‐test on the normalized gene counts. Statistical significance was defined by a threshold of *p* < 0.05. To confirm the effect of IAA‐AHR signaling on the mRNA levels of intestinal barrier‐associated genes, the relative mRNA expression levels of these genes were further validated by qPCR using gene‐specific primers (Table S5).

### Histopathology

Colon samples were harvested following mouse euthanasia, briefly washed with ice‐cold PBS, and immersed in 4% paraformaldehyde at 4°C for 24 h. Fixed tissues were processed by sequential dehydration in graded ethanol, xylene clearing, paraffin embedding, and sectioning at a thickness of 4 μm. Goblet cell distribution was evaluated using AB‐PAS staining, with hematoxylin applied for nuclear counterstaining. For morphometric analysis, three randomly chosen, non‐overlapping microscopic fields were analyzed per section. Goblet cell numbers per crypt were manually quantified using ImageJ software (NIH).

### Immunofluorescence staining

Cells grown on glass coverslips were fixed in 4% paraformaldehyde for 20 min, permeabilized with 0.1% Triton X‐100 in PBS for 15 min, and blocked in 5% BSA/PBS for 1 h at room temperature. Primary antibodies were diluted in 1% BSA/PBS and incubated overnight at 4°C at the following working concentrations (coverslips): anti‐γH2AX (1:500, Proteintech), anti‐P16^INK4a^ (1:200, Proteintech), and anti‐CLDN10 (1:500, Proteintech). After washing, Fam‐ or Cy5‐conjugated secondary antibodies (anti‐mouse/anti‐rabbit) were applied at 1:1000 for 1 h at room temperature, followed by DAPI (1 µg/mL, 5 min). Formalin‐fixed paraffin‐embedded colon sections were deparaffinized, rehydrated, and subjected to heat‐mediated antigen retrieval (10 mM citrate buffer, pH 6.0, 95–98°C, 15 min), then processed as above for blocking, primary (same dilutions), and secondary antibody incubations. Images were acquired on a confocal microscope (FV10i, Olympus) under identical exposure settings. Fluorescence intensities for γH2AX, P16, and CLDN10 in colon sections and Caco‐2 coverslips were quantified in ImageJ software (NIH) after background subtraction brightness/contrast, and levels were adjusted uniformly across groups for visualization only.

### Statistical analysis

Statistical data analysis was performed using GraphPad Prism software (version 8.0). Correlation network analyses were conducted using R v4.4.2. Data were presented as mean ± standard deviation (SD). Normality was assessed with the Shapiro–Wilk test. For two‐group comparisons, unpaired two‐tailed Student's *t*‐tests were used for normally distributed data and Mann–Whitney *U* tests for non‐normal data. Unless otherwise stated, for comparisons among multiple groups, the Kruskal–Wallis test followed by Dunn's multiple comparisons posttest was performed. The *p*‐value < 0.05 was considered statistically significant.

## AUTHOR CONTRIBUTIONS

**Wei‐Chuan Lin**: Conceptualization; methodology; data curation; formal analysis; investigation; validation; visualization; writing—original draft; writing—review and editing. **Cui Zhang**: Methodology; data curation; investigation; formal analysis; visualization. **He‐Hua Lei**: Methodology; data curation; formal analysis; investigation; writing—review and editing. **Zheng Cao**: Methodology; formal analysis; validation. **Xin Gao**: Methodology; formal analysis. **Wen‐Kai Yu**: Methodology; formal analysis. **Xin‐Zhi Li**: Methodology; writing—review and editing. **Qing‐Wei Xiang**: Methodology; writing—review and editing; resources. **Zhi‐Wen Zhang**: Methodology; resources; writing—review and editing. **Shi‐Fu Pang**: Writing—review and editing; methodology; resources. **Wei‐Fei Luo**: Supervision; methodology. **Deng‐Hui Xie**: Supervision; methodology; writing—review and editing; resources. **Li‐Min Zhang**: Supervision; conceptualization; methodology; data curation; investigation; validation; writing—original draft; writing—review and editing; funding acquisition; project administration. **Gang Chen**: Supervision; methodology; data curation; investigation; writing—review and editing; funding acquisition; resources; project administration. All authors have read the final manuscript and approved it for publication.

## CONFLICT OF INTEREST STATEMENT

Wei‐Fei Luo and Shi‐Fu Pang are shareholders and employees of AIage Life Science Corporation. AIage Life Science Corporation partially funded this research. The remaining authors declare no conflicts of interest.

## ETHICS STATEMENT

The study protocol was reviewed and approved by the Ethics Committee of AIage Life Science Corporation (No. AS‐LL‐ZD‐001) and the Ethics Committee of the First Affiliated Hospital of Guangxi Medical University (No. 2020‐KT‐050). All animal procedures were authorized by the Animal Ethics Committee of the Innovation Academy for Precision Measurement Science and Technology, CAS (No. APM24005A).

## Supporting information


**Figure S1.** Microbial diversity and feature ASVs of gut microbiome in centenarians.
**Figure S2.** Topological attributions of co‐occurrence network among young, older, and centenarian groups.
**Figure S3.** Metabolomic profiling revealed distinct patterns of age‐associated metabolic in human cohorts.

**Table S1.** Permutational multivariate analysis of variance for host variables affecting intestinal bacterial community using Bray–Curtis distance.
**Table S2.** Keystone taxa in intestinal bacteria co‐occurrence network in young, older, and centenarian cohorts.
**Table S3.** Keystone taxa in intestinal bacteria co‐occurrence network in aged mice after *C. scindens* supplementation.
**Table S4.** Primers used for PCR of *aldh* and *amiE* in *C. scindens*.
**Table S5.** Primers used for RT‐qPCR.
**Table S6.** Metadata information for the human dataset in this study.

## Data Availability

The data that support the findings of this study are available on request from the corresponding author. The data are not publicly available due to privacy or ethical restrictions. Due to ethical and legal restrictions, raw sequencing data of the human intestinal microbiotal and metabolomics datasets cannot be made publicly available. Human metadata information is available in Table S6. The other relevant data generated during the current study are available from the corresponding author upon reasonable request, subject to applicable ethical and legal approvals. The raw microbiota data and mouse intestinal RNA sequencing data have been deposited in the National Center for Biotechnology Information GenBank repository under BioProject accession numbers PRJNA1456507 (https://www.ncbi.nlm.nih.gov/bioproject/PRJNA1456507) and PRJNA1344669 (https://www.ncbi.nlm.nih.gov/bioproject/PRJNA1344669), respectively. The data and scripts used are saved in GitHub https://github.com/WeichuanLin6688/Microbial-keystone-taxa-contribute-to-intestinal-homeostasis. Supplementary materials (figures, tables, graphical abstract, slides, videos, Chinese translated version, and updated materials) can be found in the online DOI or iMeta Science http://www.imeta.science/.
