# Supplementary material for: Microbial keystone taxa and metabolic signatures in centenarians regulate intestinal homeostasis during aging
Source: Imeta. 2026 May 19;5(3):e70134. doi: 10.1002/imt2.70134 (PMC13377416; doi:10.1002/imt2.70134)
Supplement: Supplementary file 1 — Figure S1. Microbial diversity and feature ASVs of gut microbiome in centenarians. Figure S2. Topological attributions of co‐occurrence network among young, older, and centenarian groups. Figure S3. Metabolomic profiling revealed distinct patterns of age‐associated metabolic in human cohorts. [file IMT2-5-e70134-s001.docx]

**Supporting Information to**

**Microbial keystone taxa and metabolic signatures in centenarians regulate intestinal homeostasis during aging**

**Running title:** Microbial keystone taxa contribute to intestinal homeostasis

Wei-Chuan Lin^1#^, Cui Zhang^1,2#^, He-Hua Lei^1,2#^, Zheng Cao^1,2^, Xin Gao^1^, Wen-Kai Yu^1,2^, Xin-Zhi Li^3^, Qing-Wei Xiang^4^, Zhi-Wen Zhang^4^, Shi-Fu Pang^5^, Wei-Fei Luo^5*^, Deng-Hui Xie^6*^, Li-Min Zhang^1,2*^, Gang Chen^4*^

^1^State Key Laboratory of Magnetic Resonance and Imaging, National Centre for Magnetic Resonance in Wuhan, Innovation Academy of Precision Measurement Science and Technology, Chinese Academy of Sciences (CAS), Wuhan 430071, China;

^2^University of Chinese Academy of Sciences, Beijing 100049, China;

^3^School of Pharmacy, Faculty of Medicine, Laboratory for Drug Discovery from Natural Resource, State Key Laboratory of Quality Research in Chinese Medicine, Macau University of Science and Technology, Macao 999078, China;

^4^Hubei Shizhen Laboratory, Department of Geriatrics & Department of Orthopedic Surgery, Hubei Provincial Hospital of Traditional Chinese Medicine (Affiliated Hospital of Hubei University of Chinese Medicine), Wuhan 430060, China;

^5^Institute of Biological Science and Technology, Guangxi Academy of Sciences; Guangxi Key Laboratory of Longevity Science and Technology, AIage Life Science Corporation Ltd., Nanning 530200, China;

^6^Department of Joint Surgery, Center for Orthopaedic Surgery, The Third Affiliated Hospital of Southern Medical University, Guangzhou 510515, China;

^#^These authors contributed equally: Wei-Chuan Lin, Cui Zhang, He-Hua Lei.

*Correspondence: [Luoweifei@gxas.cn](mailto:Luoweifei@gxas.cn) (Wei-Fei Luo), [xiedenghui221122@smu.edu.cn](mailto:xiedenghui221122@smu.edu.cn) (Deng-Hui Xie), zhanglm@wipm.ac.cn (Li-Min Zhang, Lead Contact), chengang12@hbucm.edu.cn (Gang Chen)


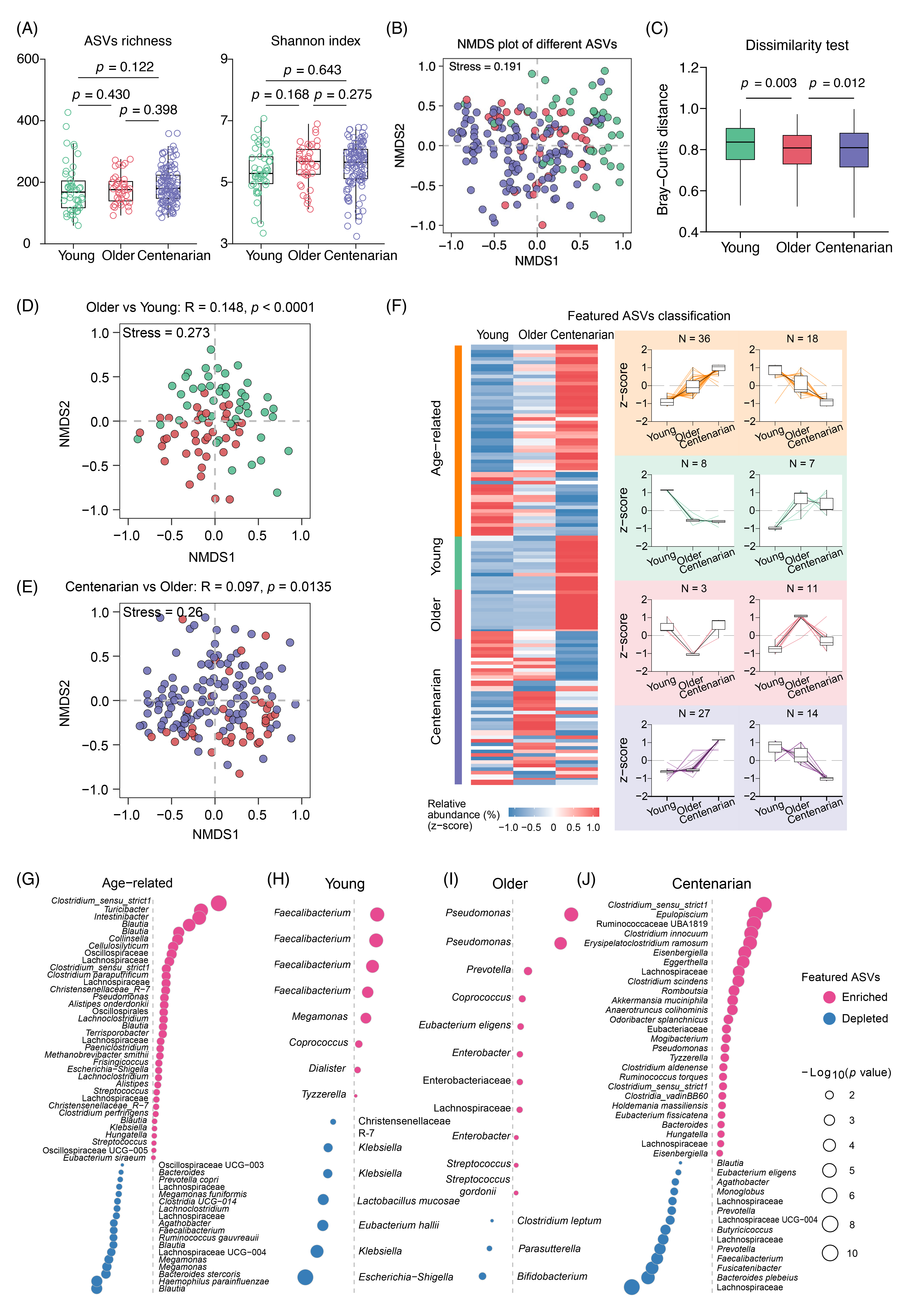


**Figure S1 Microbial diversity and feature ASVs of gut microbiome in centenarians.** (A) Differences in ASVs richness and Shannon index. (B) NMDS plots based on Bray-Curtis distance of gut microbial dissimilarity among young (green), older (red), and centenarian (purple) groups. Stress values on the left top of plot indicate the model fit. (C) Differences in the dissimilarity from Bray-Curtis distance among groups. (D) As in (B), for young (green) and older (red) groups. (E) As in (B), for older (red) and centenarian (purple) groups. (F) Classification of featured ASVs. Differential ASVs between groups were first identified by Kruskal–Wallis tests (*p* < 0.05) and then grouped into four classes: (I) age-related ASVs showing progressive trajectories across age groups, and (II–IV) group-specific ASVs uniquely enriched or depleted in the young, older, or centenarian cohorts. Relative abundances (left) and numbers (right) of features ASVs in four classes are shown. (G-J) Annotations of featured ASVs. Dot color represents enriched (pink) or depleted (blue) ASVs. Dot size represents the *p*-value (-Log transformation) from statistical significance.

**
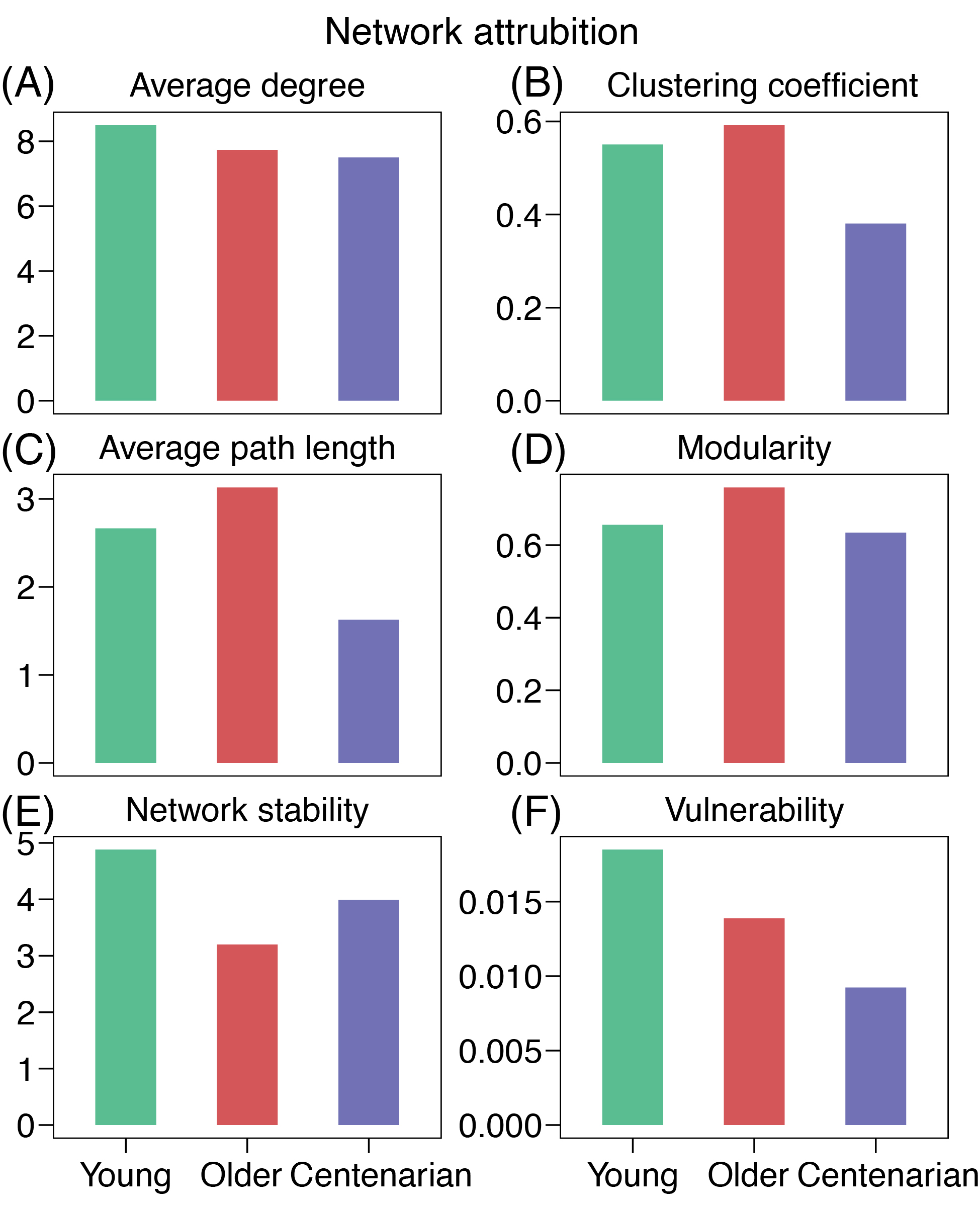
**

**Figure S2 Topological attributions of co-occurrence network among young, older, and centenarian groups.** (A) Average degree. (B) Clustering coefficient. (C) Average path length. (D) Modularity. (E) Network stability. (F) Vulnerability.


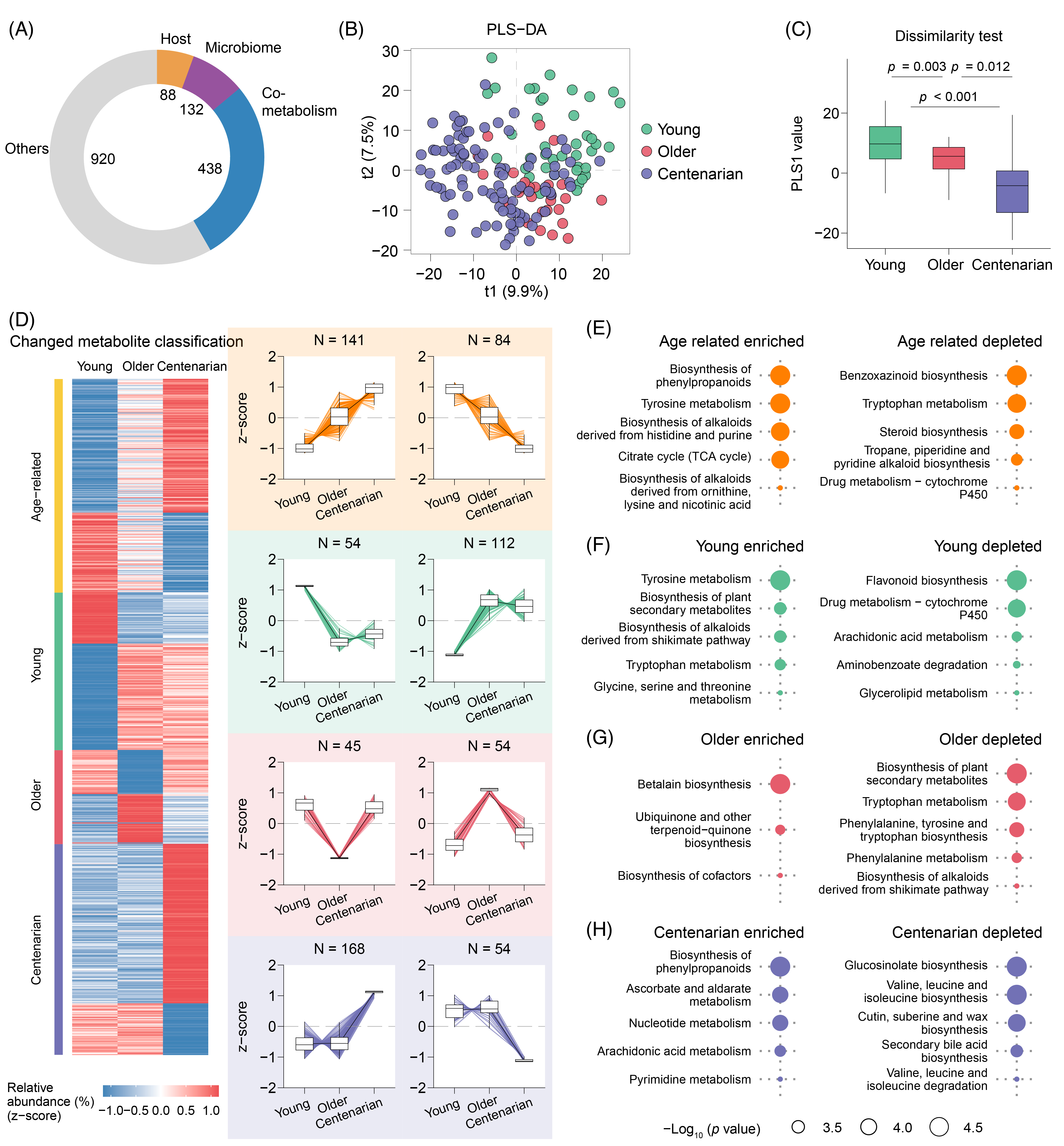


**Figure S3 Metabolomic profiling revealed distinct patterns of age-associated metabolic in human cohorts.** (A) Classification of detected metabolites from untargeted metabolome. All annotated metabolites were categorized into four major sources: host-derived (orange), microbiome-derived (purple), host-microbe co-metabolism (blue), and others (gray). (B) Partial least squares discriminant analysis (PLS-DA) of detected metabolites. The first two components (t1 and t2) from PLS-DA capture the main sources of metabolic variance. Color represents the young (green), older (red), and centenarian (blue) groups, respectively. (C) Dissimilarity test of PLS1 scores. (D) Classification of featured metabolites. Differential metabolites between groups were first identified by Kruskal–Wallis tests (*p* < 0.05) and then grouped into four classes: (I) age-related metabolites s showing progressive trajectories across age groups, and (II–IV) group-specific metabolites uniquely enriched or depleted in the young, older, or centenarian cohorts. Relative abundances (left) and numbers (right) of features metabolites in four classes are shown. (E-H) Top 5 enriched/depleted pathways from featured metabolites in four classes. Dot size represents the *p*-value (-Log transformation) from KEGG enrichment analysis.
